# Reflective thinking and medical students: some thoughtful distillations regarding John Dewey and Hannah Arendt

**DOI:** 10.1186/1747-5341-4-5

**Published:** 2009-04-16

**Authors:** Thomas J Papadimos

**Affiliations:** 1 University of Toledo College of Medicine, Department of Anesthesiology, 3000 Arlington Avenue, Toledo, Ohio 43614, USA; 2 University of Michigan, School of Medicine, Department of Anesthesiology, 1500 East Medical Center Drive, Ann Arbor, MI 48109, USA

## Abstract

Reflective thought (critical thinking) is essential to the medical student who hopes to become an effective physician. John Dewey, one of America's foremost educators in the early twentieth century, revolutionized critical thinking and its role in education. In the mid twentieth century Hannah Arendt provided profound insights into the problem of diminishing human agency and political freedom. Taken together, Dewey's insight regarding reflective thought, and Arendt's view of action, speech, and power in the public realm, provide mentors and teachers of medical students guidance in the training of thought and the need for its effective projection at the patient's bedside and in the community.

## Background

Reflective thought (critical thinking), education, human agency, social freedoms, and responsibility are all important topics of discussion in the tutelage of future physicians. John Dewey (1859–1952) was one of America's foremost educators and philosophers. His work, *How We Think*, revolutionized critical thinking and its role in education with its release in 1910 [1]. He defined reflective thinking as "active, persistent and careful consideration of any belief or supposed form of knowledge in the light of the grounds that support it, and the further conclusion to which it tends" [1].

Hannah Arendt (1906–1975) published *The Human Condition *in 1958 and provided profound insights into the problem of "diminishing human agency and political freedom; the paradox that as human powers increase through technological and humanistic inquiry, we are less equipped to control the consequences of our actions" [2]. Her thoughts still ring true today.

The thoughts of Dewey and Arendt seem to have a natural association in today's medical school environment vis a vis the pressures exerted by the environment external to academia (institutions, government, and industry, etc.), and sometimes even from academia itself. How so? Dewey explains that reflection is the discomfort students experience when they realize their grasp of a topic is inadequate [1], and this unease is amplified when students are faced with their ethical duty in the face of authority, as exemplified by the work of Arendt [2,3]. Reflective thought is crucial in the face of today's healthcare economic realities, especially in view of the pressure to compromise care that is brought to bear on practitioners by other concerned stakeholders (insurers, government, pharmaceuticals, and hospital administrators, etc.). While reflective learning has become increasingly popular [4-7], very little has been published in regard to its application to medical students [7-9]. The public-at-large and academic experts outside of medicine may find it difficult to believe that reflective learning may not regularly occur in medical education, this may indeed be the case. First, reflective thinking/learning may not be taught at all. If it is taught, it may be taught ineffectively. Such tenets may be difficult to document. However, researchers involved with today's medical education have found that students will not take up reflective learning voluntarily if they do not perceive it is related to their assignments or curriculum [7]. Also, it has been posited that both mentors and students feel that medical student's self-assessment of personal and professional behaviors to be difficult [10]. So, if reflective thinking/learning is taught ineffectively, (1) it may not be valued by the students, (2) they may not be good at it, and/or (3) they may not be capable of making an accurate self-assessment of its role in their education and future practice. Therefore, if medical students do not value reflective learning for its intrinsic value to their thought processes regarding inquiry and, in turn, cannot assess behaviors important to their well being, then it is even more imperative that inquiry through reflective thinking be inculcated in medical students through the medical faculty. Fortunately it has been demonstrated that medical schools can exert a great influence on students in regard to the need for reflective learning [7]. The General Medical Council (UK) [11] and the American Board of Internal Medicine [12] have indicated that practitioners should be able to evaluate their effectiveness and capabilities through informed criticism. If reflective learning is not occurring, or is not effective, then these tasks of personal accounting will not take place, and if they do, they may not be honest or effective.

This commentary will dwell on Dewey's concern about the problem of the training of thought and Arendt's view that we disclose our own image (as humans) through speech and action in the space of appearance, and how these thoughts are important and relevant to the mentoring of medical students in regard to reflective thought. Dewey was keen on pointing out that students need to know how to think, and Arendt enthusiastically argued that action and speech revealed unique distinctiveness (especially regarding ethicality in the face of authority). Medical students need to be astute thinkers, and, as physicians, their "art" will provide them with "distinctiveness" in the community where their thought and speech will be very important in relation to their actions.

If students will not take up reflective learning unless it is related to a personal academic benefit, and if little is published regarding reflective thought, or learning, in medical education, one may surmise, rightly or wrongly, that reflective thinking is (a) not taught or role-modeled by teachers/mentors, (b) today's teachers/mentors do not engage in reflective thinking, (c) if they do, they do not impart such knowledge, and/or (d) such reflective thinking and learning is not valued by teachers/mentors. While most observers would acknowledge, instinctively, the value of reflective thought/learning, the lack of its emphasis in medical education is arguable. The thoughts of Dewey and Arendt may help provide insight to medical educators trying to foster habits of reflective thinking in their trainees. While most observers may acknowledge its value, they may not understand the source of its value. Dewey's and Arendt's thoughts help us to understand this source.

## Discussion

### Why distill the thoughts of Dewey and Arendt?

When it comes to an education in a democracy it is difficult to argue against the primary, major influences of Kant, Dewey, and Arendt [13]. While Kant's idea of rational autonomy still influences liberal arts education today, its individual and rational perspective has received criticism from Nietzsche, Foucault, and others because it confines its position to the subject's ability to provide internal, rational control, whereas Dewey and Arendt focus on interaction in a social environment where a two-way exchange of interactions occurs between subjects and their environment, institutions, etc. Kant's views regarding rational autonomy are conceded, but there are societal discourses afoot in the 21^st ^century (health of the un- or underinsured, malpractice, cloning, pharmaceuticals, end-of-life considerations, rationing of scarce resources, etc.) which may affect the practicing physician that are better confronted using the social and political subjectivity models of Dewey and Arendt regarding reflective thinking.

According to Dewey, the "mind" is something that is acquired and, thus, students receive an education set by social conditions [14]. Education is the result of unlimited interactions that occur in a social medium between all subjects. There are no "senders and receivers" of information according to Dewey, it is always a two-way street [13]. Learning is a cooperative activity that is regulated by social partnerships [13]. Furthermore, Dewey teaches that the ability to reflect is not innate; the ability to think and reflect has a social origin, i.e., the social conception of subjectivity [13].

People evolve through social interactions, "securing direction and development in the immature through their participation in the life of the group to which they belong." [15]. There are many interests among students and faculty, therefore an important and effective interplay occurs in this particular social medium. Different interests are constantly exchanged or shared. Each individual's actions are in reference to social interplay. Dewey argues for social intelligence, in other words, intelligent cooperation [13] in that people produce and manage social institutions. Social interaction liberates humans to operate at their full capacity and develop social intelligence, thereby participating successfully in their immediate environment and in life, generally.

While thinking and reflection are of paramount consideration to Arendt, the actions of humans are even a more necessary adjunct to the human condition in her opinion. Dewey's social concept of subjectivity was important and original to Arendt, but Arendt adds the political concept of subjectivity, "the main reason for this is that Arendt holds that my subjectivity is only possible in the situation in which others can be subjects as well" [13]. For Arendt action is not isolated, others are needed, i.e., plurality. A human creates an initiative and other humans respond to this action, leading to other actions. Arendt's profound addition to educational philosophy is her observation that the human response to particular actions is unpredictable, and the responses and outcomes from individual human actions/interactions are innumerable [13]. Thus, the possibilities for the human future are infinite.

Therefore, Dewey's theory of inquiry (see below), applied through intelligent cooperation (social subjectivity), when linked with Arendt's political concept of subjectivity associated with action, provides a continuum of the evolution of thought regarding reflective thinking.

### Dewey and reflective thought

It is difficult to speak to Dewey's ideas regarding reflective thought without mentioning his ideas regarding his theory of inquiry. Burke summarized Dewey's theory succinctly [16]. The linkage between Dewey's views on reflective thought and inquiry is inseparable and must be acknowledged, although we cannot give it a full airing in this paper, it can be summarized as follows.

A conviction (belief) as a thought needs to be supported by evidence. The conviction begins as an indeterminate state that is qualitative in nature. Inquiry is a set of operations used to discover conditions that will describe the problem (conviction). The conditions need to be observed (experimentally). Thereby a student may be able to state the conditions of the problem. Once the conditions are stated further observations are made until the problem assumes a "form" that suggests a solution, or solutions. Reasoning is used to elaborate on the possible meanings of the observations (or data). Inferences then may be made. Inferences lead to "warranted assertions" [16] in regard to attaining knowledge (inferences are not knowledge). This is followed by new observations/experiments for use in proving or disproving old or new hypotheses. These modifications will continue until a "determinate situation is instituted" [13]. Reflective thought leads to inquiry that will help a physician/student arrive at a determinate situation. Dewey's thoughts on the importance of inquiry and reflective thought dovetail with Arendt's views on action, speech, power and the space of appearance.

According to Dewey "thought" may be viewed from 3 vantage points, a "broadest" vantage point to a more restricted view of its meaning [17]. In its loosest sense it is merely the ability to be aware of anything that enters our consciousness. A second, more restricted tier involves the contention that thought cannot be directly seen, heard, smelled, or tasted. In its most restricted sense a thought is a conviction (belief) that should be supported by "evidence or testimony". This third point has two prongs. In the first case, a belief may be adhered to with no evidence of support. In the second case,

...the ground or bias for a belief is deliberately sought and its adequacy to support the belief examined. This process is called *reflective thought*; it alone is truly educative in value... [17].

It is reflective thought that needs training and encouragement. Dewey makes it abundantly clear that daydreams do not count as thoughts. Just having a sequence of ideas is not enough. In reflective thought a sequence of thoughts results in a consequence, "a consecutive ordering in such a way that each determines the next as its proper outcome, while each in turn leans back on its predecessors" [17]. Each individual idea, or thought, leads to the next. The predecessor is the basis for the successor. Thus leading to a "thread" of thinking.

The student creates a belief or idea, in reflective thought, based upon a premise rooted in knowledge that goes beyond what has been presented to him or her. This is notable because the student is now accepting or rejecting something "as reasonable, probable, or improbable" [17]. Dewey recommends caution here because acceptance or rejection of an idea needs evidence: "Some beliefs are accepted when their grounds have not themselves been considered, others are accepted because their grounds have been examined" [17].

Dewey's thoughts have provided us with a basis for making the distinction between the anecdote in medical practice and evidence-based medicine. There can be no "supposition accepted without reference to its real grounds" [17]. When a physician teacher/mentor tries to teach from experience, many times prejudices, or biases, may be involved. Comments such as "I have always done it this way", or "In my experience..." are thoughts or ideas, but they may not be "judgments proper that rest upon a survey of the evidence" [17]. While there is value in personal experience, "values with reference to the support they afford the belief has not been considered" [17]. When a student's ideas (thoughts) become important to him or her, leading them to reflective thought (a chain of thoughts that have consequences that are rooted in the examination of the chain of thoughts in question), then something significant has occurred. However, it should not only be that their thoughts are important, but also those of their patients. When a student engages in reflective thought, not only on their own behalf, but also taking into consideration the patient's circumstance, then teachers, role models, and mentors have been successful.

Previously "reflective" predecessors took risks in believing the world was not flat and that the sun did not revolve around the earth. Such reflective thought needs encouragement. Students must be able to question their mentors' and teachers' beliefs. A mentor should instill an element of "doubtful inquisition" into students so that they can examine previously reasoned conclusions. Students must be able to examine their beliefs and knowledge through honest inquiry with the evidence available, regardless of what conclusions may be formulated. This constitutes reflective thought [17].

Mentors and teachers should encourage Dewey's central factor in critical thinking, that is, in viewing a set of circumstances, or a situation, that there is a "suggestion of something not observed" [17]. For example, at the bedside of a critically ill patient, a drop in the number of platelets and a rise in the white blood cell count are suggestive of sepsis. When a situation, or set of conditions, suggests a conclusion or belief, "it possesses the quality of evidence" [17]. An example of something that was suggestive, but not observed on the societal level until recently (over the last decade or so), was the potential for an adverse community healthcare outcome reflected by the large number of people without health insurance (> 45,000,000) [18]. Their condition should portend a situation in which this group has a higher morbidity and mortality than those insured, which in turn would place a financial strain on the rest of society through shifted costs and lost tax revenues. Therefore,

This function by which one thing signifies or indicates another, and thereby leads us to consider how far one may be regarded as warrant for belief in the other, is then the central factor in all reflective or distinctly intellectual thinking...Reflection thus implies that something is believed in (or disbelieved in), not on its own direct accord, but through something else which stands as witness, evidence, proof, voucher, warrant; this is, as ground of belief [17].

Reflective or distinctly intellectual thinking is absolutely necessary in the course of patient diagnoses and scientific inquiry. Mentors and teachers need to encourage such a thought process so students understand that certain sets of circumstances may also lead to desirable outcomes outside the hospital, i.e., the community. In other words, the thoughts and actions of physicians and their representative organizations may lead to social and political consequences, either as resolutions (or exacerbations). For example, the American Medical Association's current initiative regarding the need for health insurance for all is an outgrowth of reflective or distinctly intellectual thinking [18]. The reflection and belief in evidence, "in which present facts suggest other facts (or truths) in such a way as to induce belief in the latter upon the ground of the former" [17] is something that needs to be encouraged in medical school, especially in how it affects the outcomes of health in society, or an individual. The point here is that reflective thought requires one to look beyond the obvious. It is important to probe deeply, inquire thoroughly, reflect thoughtfully, and infer appropriately.

Dewey encourages mentors and teachers to be patient with the perplexity, hesitation, and doubt exhibited by students because this will lead them to a search for facts that will corroborate beliefs and conclusions. Mentors should nurture them and be tolerant during these learning exercises/situations. They need to be supported as they work through "forked-road" conditions in trying to discover truths because "demand for the solution of a perplexity is the steadying and guiding factor in the process of reflection" [17].

Are mentors truly important in this process of thinking and reflection? Of course they are! The student is faced with a problem, and because of his or her inexperience the student may not be able to form the answer. Dewey writes that the answer can only be suggested (as a starting point). He claims the student's past experience will help in supplying a suggested conclusion [17]. Such a thought can be taken one step further. Mentors can clearly be the sources of suggestion, or at least alternative sources of suggestion. Past experiences and prior knowledge can be brought to bear on a perplexing clinical or social problem (hopefully through inquiry that is based on evidence). The support and nurturing of students by mentors during an honest inquiry into a question of interest is crucial. Not only does such an inquiry require the suspension of judgment, but also the maintenance of doubt [17]. Maintaining such a position could be difficult for a student and may require the close support of a trusted mentor or advisor.

### Dewey and thought training

Dewey was emphatic that thought needed training because it was of value; quite an understatement, but why is thought of value? Dewey raises three important points regarding the value of thought [19].

First, "a thinking being can, accordingly, act on the basis of the absent and the future" [19]. It is important to realize that "thought affords the sole method of escape from purely impulsive or purely routine action" [19] in that, reflective thought arises when our impulses and habits are blocked. For example, influenza killed many people in the early part of the twentieth century, ergo vaccines were needed; sepsis, if recognized and treated early with fluids, antibiotics, and vasopressors (if necessary) may save many lives, ergo the international sepsis campaign; and rebuilding the infrastructure and economies of enemies defeated in war (Germany, Japan, Iraq), is in the best interest of the conquerors.

Second, by using thought humans set up early warning systems for their species. Humans have the capability of "systematized foresight", in that "by thought man also develops and arranges artificial signs to remind him in advance of consequences, and ways to securing and avoiding them" [19]. For example, physicians perform extensive patient monitoring in intensive care units, Doppler radar warns of dangerous weather patterns, home security systems detect intruders, highways are built with road signs, and automobiles use navigational devices and built in phone systems.

Third, "thought confers upon physical events and objects a very different status and value from that which they possess to a being that does not reflect" [19]. In other words, a well-schooled mind can draw inferences. A student must understand the facts or evidence presented, and make conclusions that lead to appropriate action. Dewey indicates that John Stuart Mill was concise and insightful in regard to this matter [20]. Drawing inferences is exceptionally important in life. Every person has a need to continually assess facts that cannot be observed, and are not directly available. These facts are important to one's daily endeavors, or "the business of life" [20]. Proper inferences require inquiry, arrived at through reflective thought, and ought to lead to beneficial action for an individual or a society.

Drawing proper inferences is important in medical, scientific, economic, political, and social circumstances. Faculty cannot ensure that students will always have the correct answer in all situations, but they can make sure that students "doubt" appropriately. Dewey argues that this thinking process needs safeguarding and training because, "an operation of drawing inferences, of basing conclusions upon evidence, of reaching belief indirectly, it is an operation that may go wrong as well as right" [19].

While educators do not have to prove every statement or teach every fact available, it is important that teachers/mentors cultivate keen habits of discrimination that are not merely opinions. Teachers must help students develop a well-grounded methodology of inquiry and reasoning that is appropriated to the problem that confronts them [19].

The ability to come to an intelligent conclusion regarding a patient's condition is important, but the ability of a physician to contribute to solving the difficult problems in a society through intelligent dialogue, thoughtful reflection, and reaching the proper conclusions by acting on the basis of the absent in regard to the future, with the support of systematized foresight that leads to the drawing of proper inferences, is a professional attribute of immense value to the community. Thus, the prepared, reflective mind of the medical student allows him or her to become an effective physician, not only as a healer, but also as a human who can effect change for the betterment of others through action in society's "space of appearance" [21].

### Reflective thought in medical education

While, on the one hand, we have contended that reflective thought and learning are necessary in medical education, a seemingly trite and obvious statement, we have also demonstrated that there is little research published regarding this contention. If this is so, then our system of medical education may be in jeopardy because of the erroneous assumption that reflective thought in our traditional system of medical education is valued, when in fact it may not be. It seems to be that medical education is neither set up to teach reflective thought/learning nor set up to challenge this view about the primacy of the facts.

If we know that reflective thought can be fostered psychologically, socially, and educationally, where are there concrete examples of its valued use to a medical student? Reflective thought can be turned "inward" and "outward".

By "inward" we mean its use in personal epistemology, referring what medical students know about knowing, "what knowledge is and how knowing is justified" [22]. Knight and Mattick have stated that in this time of evidence-based medicine (EBM) there needs to be a high level of sophistication to appreciate both the scientific and the patient vantage points in clinical medicine because there are limits to scientific facts and methodology [22], and Barbour has indicted that qualitative research results need to be incorporated into the quantitative findings [23]. So when qualitative factors are brought together with quantitative factors "truth" becomes viewed from different angles and may leave the impression that truth/knowledge is being constructed [22]. Here the term "constructed' is used in the Foucaultian sense in that prevailing discourses in a society are usually directed and perpetuated by powerful stakeholders [24]. Therefore, reflective thought becomes of paramount importance in regard to how facts (truth and knowledge) are formulated, presented, applied, and by whom.

By the term "outward" we mean the ability to use reflective thought, as a physician, in questioning healthcare stakeholders outside of the individual patient, such as insurers, healthcare administrators, government, and other regulating bodies (inside and outside of academia). Again, we will use the example of EBM, but not as it applies to the individual patient, and without taking a stance among EBM protagonists and antagonists. There is a debate going on between two camps as whether or not we can "situate EBM in relation to theory and practice, demonstrating that, without critical theoretical insight into the epistemological and political assumptions that underpin the logic of EBM, EBM amounts to an unethical and dangerous practice" [25]. This debate exemplifies the need for reflective thought and learning when dealing with a controversial and complicated topic. Therefore, forms of education that instill habits conducive to independent and critical thinking as opposed to rigidity of the thought are important. If students acquire good habits regarding their education then reflective thinking may become habitual [26,27].

### Reflective thought and Arendt's action, speech, power, and space of appearance

Reflective thought, refined in medical students, allows them to become physicians who can make appropriate inferences regarding the human condition (medical and social) by drawing on the past, the potential of the unknown future, and the abstract. Thus, making the correct inference will lead to action. While human plurality is, "the basic condition of both action and speech" [21], reflective thought is required to support this speech and action. Human plurality consists of equality and distinction. All humans are equal and each human is uniquely distinct,

Speech and action reveal this unique distinctness. Through them men distinguish themselves instead of being merely distinctive; they are the modes in which human beings appear to each other, not indeed as physical objects, but *qua *men [21].

Reflective thought is necessary for the promotion of equality and distinctiveness. Without reflective thought we may be left with moral standards and norms that are unreliable and interchangeable [25]. So what does reflective thought provide that might make moral standards and norms reliable? The answer to this question is linked to Arendt's view of Adolf Eichman's inability to think and have an internal dialogue with his "self" (during the Jewish holocaust), as interpreted through Socrates' thought on philosophy and personal integrity [28]. Arendt explained that thinking involved endless dialogue with one's self and that there were moral implications with this process. This endless dialogue, or "thinking", needs to occur in the solitude of a person's mind, far from the hustle and bustle of everyday life [28], but "in spite of the efforts of philosophers ever since Plato to extract solid results such as moral rules from the process of thinking, the mind's internal dialogue does not produce anything at all, and is more likely to undermine accepted rules with its incessant questioning" [28]. However, having a dialogue with one's "self" may place limits on the behavior of a person. Socrates' view, in a nutshell, was that "it was better to be wronged than to do wrong" because he had to go on living with himself with the dialogue of his own mind [28]. According to Arendt such "consciousness" becomes "conscience" [28]. Therefore, reflective thought supports an endless internal dialogue with one's "self" that ought to lead to behavioral changes/corrections.

Physicians may, generally, be intelligent, but does intelligence guarantee ethicality, or correctness in decision making? Will our citizenry adopt any code that is expedient for the public welfare? Will physicians accept any code that makes their medical practice easier? Expedient codes may involve euthanasia, treating the medically needy, accepting drug trials done in vulnerable (third world) populations leading to medical breakthroughs, or donation/acquisition of organs from felons. Reflective thought through a "soundless dialogue" between a physician and his "self" cannot be overemphasized [19] because such a self-relation is *authentic thought,*

What, then, can we rely on when systems of authority are suspect, when our feelings are unreliable, and when our intelligence cannot be trusted either? For Arendt, this is a political and ethical question. And to answer it, she looks not to the way the individual relates to the external world, not to the individual's relation to others or even to a set of codes or norms; she defines this self-relation as *authentic thought *[25].

Dewey's view of reflective thought and Arendt's view of authentic thought are inextricably intertwined. The teaching of reflective thought/reflective learning may lead to the prevention of the interchangeability of moral standards and norms by providing an environment for a healthy self-relation in future physicians.

Encouraging reflective thought has an enormous educative value to a medical student's "appearance" (a socially aware and active self) in society, especially in relation to action and speech because individuals reveal their unique personality in their action and speech. Thus, they present themselves to the world around them as "who" they are in contradistinction to "what" they are. His or her unique abilities, or shortcomings, which may be hidden, are evident in everything a person says or does [21]. "Who one is", as opposed to "what one is", is necessary in the evolution of a physician's professional identity [25], especially in relation to society-at-large.

In the evolution of a physician's identity reflective thought is illuminating. Its effect on speech and action is legion, "because of its inherent tendency to disclose the agent together with the act, action needs for its full appearance the shining brightness of glory, and which is possible only in the public realm" [21]. The former medical student, now a practicing physician, who is thoughtfully reflective, has the opportunity to influence people, organizations, and institutions in society through his or her appearance in the public realm. The public realm does not necessarily mean an appearance on television, radio, in the newspapers, or a magazine, although it could. The public realm "is the space of appearance in the widest sense of the word, namely, the space where I appear to others as others appear to me, where men exist not merely like other living or inanimate things but make their appearance explicitly" [21]. This space of appearance predates governments and goes back to the beginning of interactions between men. It must have had its conception at a point in time just after the origins of reflective thought, when man put together a string of thoughts that had consequences that he expressed through action. This action then needed speech so the agent of action could convey his reasoning,

The action he begins is humanly disclosed by the word, and though his deed can be perceived in its brute, physical appearance without accompaniment, it becomes relevant only through the spoken word in which he identifies himself as the actor, announcing what he does, has done, and intends to do. No other human performance requires speech to the same extent as action 

The space of appearance occurs anywhere and anytime men and women get together through speech and action. This space disappears with the dispersal of the actors and the disappearance of the cause that brought them together [21]. However, without reflective thought leading to speech and action there would be no gathering of humans in the public realm to discuss problems of consequence.

Reflective thought brought to bear on society's healthcare problems should lead to an action taken on behalf of those who cannot act to achieve a benefit that may aid their welfare. While reflective thought should lead to an appropriate action, this action may require speech. Therefore, reflective thought (a chain of thoughts that have consequences rooted in the examination of the chain of thoughts in question), whose necessity is explained through speech, may be very important in aiding successful action.

Reflective thought that leads to correct or helpful actions, whose necessity is artfully and effectively communicated, allows the critical thinker to exercise power (on behalf, hopefully, of what is good). "Power is what keeps the public realm, the potential space of appearance between acting and speaking men, in existence" [21]. Power comes into being when people act together and it disappears when people no longer interact or disperse. Speech and action lead to power, and power can allow the continued use of speech and action to a beneficial end. The critical thinker, i.e., the medical student, who engages in reflective thought, and becomes an effective healer and effective member of society, will most likely contribute to the use of power in a just and equitable fashion.

While the medical student needs to learn to be just in the use of power, the student also needs to be able to stand up to power. Arendt spent much of her career in writing and reflection upon the heinous regime of Adolf Hitler's Nazis. Arendt has tried to educate readers as to the fact that good government may not be wise government, that the medical establishment may wield the whip of "the public good" to the detriment of the weakest in a society, and that the citizenry itself may conform to a power structure that imposes its will [29-31]. This conformity of the citizenry is the ultimate subjugation of morality to the hegemonic imposition or manipulation of an authoritarian totality. In other words, to become physicians who exhibit the ability to "speak truth to power" [24] students ought to be taught through word and deed (speech and action) to wield such power, or truth, fearlessly on behalf of those who cannot. While role modeling is a very important method of conveying the concept of fearless speech, it could also be taught in plenary sessions, problems-based learning sessions, and in small groups. Physicians must not only enter Arendt's space of appearance as themselves with entreaties on their own behalf and that of their profession, but they may also have to enter this "space" on behalf of those cannot (i.e., the weakest of society) and with the understanding that such an undertaking is a risk to themselves (the physician).

Mentors and teachers in schools of medicine have a unique opportunity to mold, influence, and nurture thoughtful reflection regarding the problems that distress, not only individual patients and their families, but society at large. Mentors and teachers of medical students are uniquely positioned to influence mankind. How so? According to Arendt what saves the world from its natural ruinous course is natality,

The miracle that saves the world, the realm of human affairs, from its normal, 'natural' ruin is ultimately the fact of natality, in which the faculty of action is ontologically rooted. It is, in other words, the birth of new men and the new beginning, the action they are capable of by the virtue of being born [21].

Each year a new group of students enters medical school and each year a group graduates; mentors and teachers are party to a conception (first year students) and to a dispersal of hatchlings (fourth year students). They are fortunate and privileged to be part of this cycle of academic life in that they have an opportunity to influence new beginnings: "Men, though they must die, are not born in order to die but in order to begin" [21]. Every academic year provides an opportunity for the fostering of new beginnings regarding critical thinking and intellectual tolerance among students. Mentors and teachers have the opportunity to encourage reflective thought leading to challenge (action) by way of erudite argument (speech) that can result in consequences that improve patient care and improve the life of the community (through sustained power) to the benefit of the citizenry.

### Reflective thinking: an academic fancy or a real need?

The empiric evidence for more reflective thinking in medical education comes from two major efforts. The first is from the American Medical Association (AMA) and the second is from the Accreditation Council for Graduate Medical Education (ACGME).

The AMA Initiative to Transform Medical Education (ITME), based on input from the Ad Hoc Committee of Deans, the Committee on the Quality of Health Care in America; Institute of Medicine, and the Committee on the Health Professions Education Summit; Institute of Medicine, has identified "inadequacies in physicians' preparation to practice in an evolving health care system, including meeting increased expectations for safety, quality and teamwork. These concerns have been linked to gaps in the system of physician training" [32]. Furthermore, "ITME activities have been guided by the principle that transformation in medical education must include, but not be limited to, changes in what is taught, in where and how teaching occurs, and in how learner's attainment of the desired outcomes is assessed" [32]. Additionally, "the environments in which all trainees learn have been recognized by many as a key factor in their professional development. ITME, therefore, chose the learning environment as its first Phase 3 priority" [32]. Let there be no doubt that social subjectivity (Dewey) and political subjectivity (Arendt) play a role here.

The ACGME's Outcome Project is also of significance [33]. This project wants to move the model of accreditation of medical programs from one of *potential *to one of *actual accomplishments *regarding six core competencies that are relevant to the effective and successful practice of medicine. Reflective thinking is applicable across the spectrum of these competencies (Patient Care, Medical Knowledge, Systems-based Practice, Professionalism, Practice-based Learning and Improvement, and Interpersonal and Communication Skills). Specifically, in regard to Practice-based Learning and Improvement the, ACGME recommends "reflection" on practice and practice analysis regarding processes and behaviors. Again, social and political subjectivity come into play with the ACGME competencies, between (a) the ACGME and the institution, (b) the administration of the institution and the department chairs, (c) the chairs and their faculty, and (4) the faculty and students, residents, ancillary health personnel, and, of course, patients.

While it would be very satisfying if all of medical education existed in Gottfried Leibniz's "best of all worlds" (as he is represented by Dr. Pangloss in Votaire's *Candide*) [34], the fact remains that this is not so. Use and teaching of reflective thinking will, in fact, vary among educational settings. If reflective thinking was well entrenched throughout medical education and practice there would have been no need for the ACGME Outcomes Project or the AMA's ITME effort. The need for reflective thinking is a repetitive academic theme that has been evident and persistent since 1910.

### How can reflective thinking be interjected into medical education?

This will vary from institution to institution, but will depend on a three-pronged evaluative approach of (1) the environment, (2) the faculty, and (3) the students, with the appropriate delivery, of an appropriate product, with a measurement of its impact and effectiveness. This could be assessed against ITME or ACGME standards/recommendations, or a locally developed standard.

The evaluation of the environment will entail the size of the institution, examination of resources (financial and otherwise), its culture/tradition, integration of faculty across disciplines, and curriculum opportunities for intervention. The faculty may be assessed as to numbers, interests, availability, abilities, and location and type of practice. The students should be assessed in regard to their numbers, usual or available teaching modalities to which they are subjected, participation in mentoring programs (which may be available to all, some or none), and, again, intervention opportunities. Once the evaluation/assessment is complete, an intervention appropriate for that particular institution can be implemented.

The mode of teaching reflective thought may be delivered through problem-based learning [35], case-based learning [35], use of web-based adjuncts [36], peer teaching [37], and lectures, but one-on-one mentoring with a concerned, well-trained (in reflective methods), interested faculty member, who can add the afore-mentioned methods, may be the most effective and ideal model. However, it is very important for teachers and mentors to understand that the teaching of medical students is the teaching of adults (andragogy), and faculty should be aware of Knowles' assumptions about adult learners [38]: (1) they are intrinsically motivated, (2) readiness to learn is inherent, (3) the need to know is strong in adult learners (benefits, consequences, and risks need to be ascertained before getting into the learning situation), (4) self-concept and intellectual responsibility lend acceptance to self-directed learning, (5) past experiences are essential to learning; group discussions, simulations, and problems solving involving with peers are effective, and (6) examining relevant issues connected to real-life situations motivates adults.

## Conclusion

Mentors and teachers ought to facilitate and nurture the concept of reflective thought. Dewey's explanation of what reflective thought is, and that it needs to be trained, tolerated, and insisted upon, is enormously applicable to the medical school environment. In essence, reflective thought is critical thinking, and critical thinking is an imperative requisite to a successful medical practice. One of the difficulties with the topic of reflective thought is the dearth of research in regard to its use and application in medical education. Studies regarding its application and use in the medical school environment need to be pursued.

When medical students become physicians, who can also apply their reflective thought skills to problems outside the individual patient, they will hopefully be able to contribute to the welfare of the many through effective inquiry that leads to speech, which conveys the need for action in Arendt's "space of appearance".

## Competing interests

The author declares that they have no competing interests.

## Authors' contributions

TJP is responsible for the entire manuscript.

## Author's information

The author is an Associate Professor of Anesthesiology at the University of Toledo College of Medicine and the University of Michigan School of Medicine. He practices critical care medicine and has interests in the care of the medically needy, the philosophy of medicine, literature and medicine, the education and mentoring of students and residents, and infection control.
